# Leveraging the potential of wild food for healthy, sustainable, and equitable local food systems: learning from a transformation lab in the Western Cape region

**DOI:** 10.1007/s11625-022-01182-3

**Published:** 2022-08-10

**Authors:** Laura M. Pereira, Sandra Boatemaa Kushitor, Carolyn Cramer, Scott Drimie, Moenieba Isaacs, Rhoda Malgas, Ethel Phiri, Chimwemwe Tembo, Jenny Willis

**Affiliations:** 1 Centre for Food Policy, City University of London, London, UK; 2 Stockholm Resilience Centre, Stockholm University, Stockholm, Sweden; 3 Global Change Institute, University of the Witwatersrand, Johannesburg, South Africa; 4 Centre for Sustainability Transitions, Stellenbosch University, Stellenbosch, South Africa; 5 Department of Community Health, Ensign Global College, Kpong, Ghana; 6 Southern Africa Food Lab, Stellenbosch University, Stellenbosch, South Africa; 7 Nutrition Division, Department of Global Health, Faculty of Health and Medicine Sciences, Stellenbosch University, Stellenbosch, South Africa; 8 Institute for Poverty Land and Agrarian Studies (PLAAS), University of Western Cape, Cape Town, South Africa; 9 Department of Conservation Ecology and Entomology, Faculty of AgriSciences, Stellenbosch University, Stellenbosch, South Africa; 10 Department of Agronomy, Faculty of AgriSciences, Stellenbosch University, Stellenbosch, South Africa; 11 Institute for Social Development and Centre of Excellence in Food Security, University of the Western Cape, Cape Town, South Africa

**Keywords:** Fynbos, Healthy diets, Neglected and underutilised species, South Africa, Sustainability transformations, T-Labs, Coastal wild foods

## Abstract

Food insecurity and diet-related diseases do not only have detrimental effects to human health, but are also underpinned by food systems that are environmentally unsustainable and culturally disconnected. Ensuring access to a healthy, affordable, and sustainable diet is one of the greatest challenges facing many low- and middle-income countries such as South Africa. These challenges in accessing a diverse diet often persist despite biocultural richness. For example, South Africa is globally recognised for its rich biodiversity, an ecologically unrivalled coastline, and a rich body of traditional knowledge amongst wild-food users. In this paper, we explore the potential that coastal wild foods as neglected and underutilised species (NUS) can play in local food systems in South Africa’s Western Cape Province. Following a previously established transformation lab (T-Lab) method, here we report the observations and outcomes emerging from a two-day workshop held in May 2019 with a group of 40 actors involved in the local food system in diverse ways. Farmers, small-scale fishers, indigenous knowledge holders, representatives from non-profit organisations, chefs, bartenders, academics, activists, conservationists, and government officials were brought together with the aim of strengthening an emerging coalition of coastal wild food actors. Findings highlighted the existence of a fledgling economy for coastal wild foods, driven by high-end chefs. The T-Lab was essentially a tool of knowledge co-production around food system transformation and helped to surface deeply embedded issues on land, race, history, and culture that warrant engagement if a better food system is to emerge. In a country that is drought prone and vulnerable to climate change, a more resilient and sustainable food system is a necessity. But defining alternative governance systems to shift towards a healthier, more sustainable, and more equitable food system will require concerted effort across all stakeholders.

## Introduction

On 24th January 2022, Kenyan vegetables attained the status of UNESCO intangible culinary heritage of humanity and were added to the Register of Good Safeguarding practices (UNESCO 2022). This was thanks to a commitment made by the government in 2007 to address the decline in food diversity and the threat of modern lifestyles to traditional foodways. This listing of Kenyan vegetables adds to a collection of over 25 food-related practices, including Mexican traditional cuisine, the Senegalese national dish Ceebu jën (Thieboudienne), Japanese food known collectively as Washoku, street hawker food in Singapore, and even Nsima (porridge made from maize meal) in Malawi. The importance of local cuisines, their associated cultural practices and knowledge, and the food system diversity that they promote are thus increasingly being recognised around the world. Unfortunately, this comes largely as a response to the decline in food system diversity that is observed globally (Khoury et al. 2014). In the face of such threats, preserving biocultural diversity is central to ensure the nutrition, resilience, and adaptive capacity of indigenous peoples and local communities who collectively maintain the longest ongoing human experiences with the provision of food under environmental change; a core component of global food security (Argumedo et al. 2021).

Alternatives to the current dietary trajectory would require a food system that is both good for people and the planet, and is developed through holistic approaches to the social–ecological factors that determine local food systems. Achieving a diversity "triple win" of production system diversity, agro-biodiversity, and dietary diversity could positively impact health for the poor who are often unable to access the healthier and more diverse diets available to the wealthier segments of society (Pereira 2017; Frison and Clément 2020). One such move to advance more sustainable food systems at a global level is through the sustainable use of neglected and underutilised species (NUS) (Mbhenyane 2016, 2017; Mabhaudhi et al. 2018; Hunter et al. 2019; Akinola et al. 2020). Hunter et al. (2019: 710) describe NUS as “useful plant species which are marginalized, if not entirely ignored, by researchers, breeders and policy makers (Padulosi et al. 2013)”.

In this paper, we take a broader view of the above definition of NUS, moving beyond plant species to include terrestrial, freshwater, and marine organisms. This is done to reflect the diversity of wild coastal food resources available in the Western Cape area of South Africa, where our case study is located. In the fynbos biome of South Africa's Cape Floristic Region (CFR), terrestrial and marine NUS are gradually being recognised for their potential as climate-resilient and nutritionally dense foods that can improve food security, diversify diets, and preserve food culture (Mbhenyane 2017; Tembo-Phiri 2019; Willis 2019), as has been shown elsewhere in the world (Biodiversity International 2017). Fynbos edible species are adapted to their local natural environment and there is a rich accumulated local knowledge about their use, rendering them culturally appropriate for adoption amongst certain user groups (Botha et al. 2020; De Vynck et al. 2016a; Singels et al. 2016). In this sense NUS have the potential to alter the trajectory away from the nutrition transition currently observed in South Africa, characterised by shifts away from unrefined foods towards unhealthy foods, and thereby to spark the emergence of local food systems that offer both nutritious and healthy food and are as aspirational as Western diets (Pereira et al. 2019). Furthermore, there is the added benefit that such NUS can potentially contribute to the development of a local economy, generating jobs and livelihoods that draw on local people's knowledge and experience of local ecosystems.

A growing body of literature on useful wild-harvested food is addressing some of the existing knowledge gaps about the use value, economic contribution, governance, and conservation of edible species in South Africa (e.g. Chakona and Shackleton 2019; De Vynck et al. 2016a; Garekae and Shackleton 2020; Shackleton and Shackleton 2004). However, few studies have focused specifically on NUS, and fewer still (e.g. De Vynck et al. 2016b) have advanced beyond a narrow focus on plants. An account of how to address food system challenges in a country such as South Africa that has historical deficits and rising inequality (Satgar and Cherry 2020) and at the same time contains some of the most biodiverse hotspots in the world (Myers et al. 2000) may shed light on similar challenges elsewhere. It may also help to inform regional and international policy on NUS and their roles in enhancing food security. In this sense, a case study of coastal wild foods (of which many are NUS) in the fynbos region of South Africa can offer an enlightening example of how to navigate socio-economic, political, and cultural tensions in an attempt to preserve food system diversity through biocultural heritage.

However, as has been increasingly shown in the literature, food system transformation cannot be undertaken just through research and its dissemination. Instead, it would require far more engaged action and transdisciplinary processes that include a wide range of food system stakeholders (Rivera-Ferre et al. 2013; Hebinck et al. 2018; den Boer et al. 2021). There remains a dearth of robust examples about the methodologies and approaches that should underpin the activities of such food-related multi-actor platforms, where communities of academics, practitioners, policymakers and citizens are coming together to co-design innovative projects around shared visions and objectives (López Cifuentes et al. 2021).

Transformation labs (T-Labs) have been proposed as a transdisciplinary methodological approach appropriate for food system transformation action research (Ely et al. 2020; Scoones et al. 2020). By design, T-Labs can strongly support such as agenda, as they elevate the role of society in seeking solutions to wicked problems, establish a transdisciplinary community of practice, enable critical self-reflexivity amongst participants, seek opportunities for experimentation, confront power, exercise agency, and take responsibility for the unfolding process (Pathways Network 2021). In essence, the transdisciplinary research approaches implicit in the T-Lab methodology seek to produce transformative knowledge outside academic institutions by forming collaborative partnerships with society (Pereira et al. 2021). Previous work has shown that a T-Lab approach can be useful in bringing diverse stakeholders together to think of solutions in the context of food systems (Ely et al. 2020; Pathways Network 2021), including the fraught South African food system (Pereira et al. 2020a).

In this paper, we explore some of the critical aspects of incorporating coastal wild foods into local diets using a T-Lab approach. We select this type of transdisciplinary approach as this is neither a simple nor a linear process, and would thus require a great deal of careful negotiation, honest dialogue, and participation by a range of stakeholders. With this in mind, we document how a T-Lab held in May 2019 aimed to bring together a diverse group of 40 stakeholders involved in the local food system in diverse ways. These actors gathered for two days in Cape Town for a process aimed at strengthening the contribution of indigenous coastal foods to a more sustainable and healthy food system in the Western Cape. We build on the legacy of T-Labs in the Western Cape region, but focus on the role of coastal NUS as a potential leverage point for shifting diets towards a more sustainable and equitable food system (see Abson et al. 2017). In the next section, we unpack the context of coastal wild foods in the Western Cape Province, and we then go on to elaborate on the T-Lab as a process and method. The results from the workshop are arranged according to the thematic areas that emerged from the participatory group process. We discuss these results and, in particular, the challenges and opportunities identified by the participants in the broader context of policy interventions for more sustainable and equitable diets. We also reflect on the learnings from using T-Labs as a transdisciplinary process.

## Methods

### Methodological approach: transdisciplinarity and T-Labs

A facilitated process that enables dialogue between diverse stakeholders in a complex social–ecological system can contribute to the change required for deep social innovation and transformation of that system (Mair and Hehenberger 2014). Such a process enables dialogue, sense-making, reflection, and reflexive learning, while supporting the reframing of issues in ways that allow solutions (or attempts to experiment and transform) to be co-created and co-realised (Sharpe et al. 2016). Hence, the method we used for this process was a transformation lab or T-Lab (Charli-Joseph et al. 2018; van Zwanenberg et al. 2018; Ely et al. 2020; Pereira et al. 2020a).

Transformation labs build upon the concept of social innovation labs as carefully designed and facilitated processes aimed at supporting multi-stakeholder groups to address complex social–ecological system problems by creating "safe enough" spaces to discuss and launch innovations (Pereira et al. 2020a: 6). Furthermore, T-Labs provide experimental niches in which people with diverse backgrounds, perspectives, interests, and areas of expertise work together to understand complex systems and the problems they generate, and to identify, co-design, and prototype novel ways of addressing the underlying causes of those problems (Gryszkiewicz et al. 2017; Olsson 2017). Therefore, a T-Lab is designed to afford a diverse group the opportunity for deeper engagement around complex issues to foster innovation and transformation within social–ecological systems (Pereira et al. 2020a). By building knowledge, networks, and commitment, a T-Lab can help create a collective sense of the need for change (within and beyond the stakeholders directly involved), identify strategies for affecting change, and identify which actors have transformative power (Drimie et al. 2018; Pereira et al. 2020a). In this way, the process can develop a change strategy that tests multiple solutions, which together could solve the challenge. It can help to create early ideas of interventions, to frame the challenge, build momentum for action, find the innovators, and increase their capacity to address the challenge more effectively.

A key dimension of the coastal wild foods T-Lab was to provide a platform for participants to learn from and share with one another, while delving into the threats and opportunities related to coastal foods. As such, the T-Lab aimed to foster practical connections, mutual learning, networking, and reflections on the prevailing food system to establish opportunities to incorporate coastal wild foods and NUS equitably into the Western Cape food economy. In constituting an alternative food system, NUS and other coastal wild foods are at a disadvantage. Most actors associated with these foods do not fit well within the existing system configuration of conventional agriculture dominated by a few powerful organisations that operate within an exploitative economic system that reproduces and benefits itself (Greenberg 2017). Alternative ideas often require protective spaces, away from the adverse selection pressures created by existing systems, not only for actors to improve the performance of more sustainable practices, but also to develop new path-breaking system configurations (van Zwanenberg et al. 2018).

Experience with previous T-Labs on similar topics pointed to the need for practical outcomes that would enhance participant agency (Pereira et al. 2020a). This was done by fostering personal reflection, networking, and strategic thought to stimulate mutual learning and new connections. The objective was for participants to work together in identifying practical steps to build new practices around coastal wild foods in the Western Cape. In this instance, the T-Lab process was built on a previous set of T-Labs established in the region, also to discuss food system transformation. Therefore, there was already an interested core group of food system actors eager to be involved (Zgambo 2018). The design and facilitation of these T-Labs were undertaken by two of the authors of this paper.

Another key dimension of the T-Lab was to build from an emerging understanding of issues towards new solutions and responses. Deliberations on how to transform the food system solicited a wide range of ideas and activities. These ranged from challenging dominant structures and practices, to seeking political alignments, from lobbying for supportive policy measures to creating new flows of coastal wild foods, and from fostering shifts in consumer and cultural norms to producing new kinds of knowledge. Here, the T-Lab process design was instrumental in building a collective sense of the need for change and an understanding of where the opportunities for transformation might lie.

As the T-Lab process is inherently relational (i.e. about individuals engaging with each other over a duration of time with a clear focus), it required a critical mass of different stakeholders who needed to relate to one another, sometimes under tense circumstances (Pereira et al. 2020a). A diversity of perspectives in such processes potentially allows for the emergence of innovation when different people from different backgrounds interact to address challenges with their collective ideas and creativity (Bennett et al. 2016). Part of this is an openness to enable learning about alternative perspectives on possible solutions. This is important to open up new learning and interpretation possibilities and to explore the breadth of possible solutions and responses. However, engaging in alternative perspectives in a highly inequitable and unjust system can challenge participants to the point that discomfort may lead to overt disagreement. The T-Lab design should thus include opportunities for different voices and perspectives to be heard, as well as chances to diffuse tensions where necessary (Drimie et al. 2018). In this regard, the emergence of new ideas and relationships was anticipated as much as the likelihood of diverse perspectives that could raise tensions. These were important factors for the design, but also impact the kinds of results that can emerge from such a process as participants raise the issues that are closest to their hearts.

### Design and structure of the T-Lab on coastal wild foods

Ideally, the form that a T-Lab will take depends on the local context and the people involved. We aimed to cover a broad range of actors in the production, processing, distribution, governance, and research in the Western Cape food system. Invitations were sent to a network of food system innovators, created by the Southern African Food Lab through previous T-Labs and community engagements. The guests were asked to suggest other stakeholders involved in the coastal wild food industry using a snowball sampling method. Invitations were then sent to the suggested parties. To ensure adequate representation from the marine and fishing communities—a group crucial to the conversation on local food systems—connections were actively sought through a local NGO, Abalobi, and through researchers who had been working with coastal fishing communities in the region.

A total of 40 participants took part in the event over 2.5 days (Table 1). The participants were researchers, farmers, indigenous knowledge holders, chefs, retailers, and government officials. Although only one actor is listed as an indigenous knowledge holder, many of the representatives from the NGO coastal actor group also identified as indigenous peoples from the Western Cape. City University of London granted ethical approval for the workshop (Soc-REC/80025567/22-04-18), and all participants signed consent forms for the use of their photographs and input during the T-Lab process.

**Table 1:** Participants in the T-Lab by primary domain of expertise

Category of stakeholder	Code	Affiliation
Researchers and academics	RES1	Administrator, Stellenbosch University
	RES2	Researcher, Southern Africa Food Lab
	RES3	Researcher, Stellenbosch University
	RES4	Lecturer in Agronomy, Stellenbosch University
	RES5	Researcher, University of the Western Cape
	RES6	Administrator, Stellenbosch University
	RES7	Researcher, City University London
	RES8	Professor in Land Governance, University of the Western Cape
	RES9	Lecturer in Human Geography, University of Cape Town
	RES10	Lecturer in Ecology and Conservation, Stellenbosch University
	RES11	Population Scientist, Stellenbosch University
	RES12	Professor in Agriculture Economics, Stellenbosch University
NGO coastal actors	NGO1	Coastal actor, Masifundise Development Trust
	NGO2	Coastal actor, Masifundise Development Trust
	NGO3	Coastal actor, Abalobi
	NGO4	Coastal actor, Abalobi
	NGO5	Coastal actor, Solomon Square
	NGO6	Coastal actor, Private
	NGO7	Coastal actor, Masifundise Development Trust
	NGO8	Coastal actor, Buffeljagsbaai
	NGO9	Coastal actor, Abalobi
	NGO10	Youth advocate, Slow Food Youth Network
	NGO11	Coastal actor, Abalobi
	NGO12	Indigenous food, Making KOS
Indigenous knowledge holder	IND1	Indigenous knowledge holder, First People Indigenous
Chefs/bartenders/hoteliers	CHE1	Chef, Skinny Legs Café
	CHE2	Chef, Private chef
	CHE3	Chef, The Black Sheep
	CHE4	Bartender, Botanical Bar
	CHE5	Hotelier, The Vineyard Hotel
Farmers	FAR1	Farmer, Meuse farm
	FAR2	Farmer, Umthunzi Farming Community
	FAR3	Farmer, Grounded
	FAR4	Farmer, UbuhleBendalo Food Gardens
	FAR5	Farmer, Khaya Garden
	FAR6	Founder, Veld and Sea
Retailers	RET2	Marketer, Harvest of Hope
	RET1	Retailer, Wild Peacock
Government officers	GOV1	Public officer, Cape Nature
	GOV2	Public officer, Department of Premier, Western Cape Government

The T-Lab was conducted on 2–3 May 2019 at Food Jams (Salt River, Cape Town) and comprised two objectives: (a) to strengthen an emerging coalition or community of coastal food actors from across the system by stimulating new connections and identifying practical steps to build practice and (b) to identify practical ways to support this coalition by building on ideas, partnerships, a sense of purpose, and practical action. Participants were invited to bring samples of coastal wild food produce and products to exhibit for the duration of the workshop.

A transdisciplinary and adaptive approach was used to convene the meeting with frequent feedback and guidance asked from participants before and during the workshop. Developing an appreciation of where human agency lies within local food systems (particularly the participants' agency) and how they could support ideas to be put into practice required a diversified programme of engagements. This was partly in recognition that a rigid structure can inhibit participation (Pereira et al. 2020a). As such, the design of the T-Lab included: (a) facilitated sessions (exploring key political and policy framings), (b) guided plenary conversations and small group conversations, (c) individual-focused activities, including "speed dating", (d) personal reflection, (e) free writing or journaling, and (f) informal interactions over shared meals, refreshment and, comfort breaks; see Cramer et al. (2019) for more information on the agenda. At the end of the T-Lab, a coastal wild food social gathering, or "Food Jam", was held. Participants and guests were able to deepen connections by preparing, cooking, and sharing a portion of the meal featuring coastal wild foods. More details of some of these sessions are provided in the following section.

**Fig. 1:**
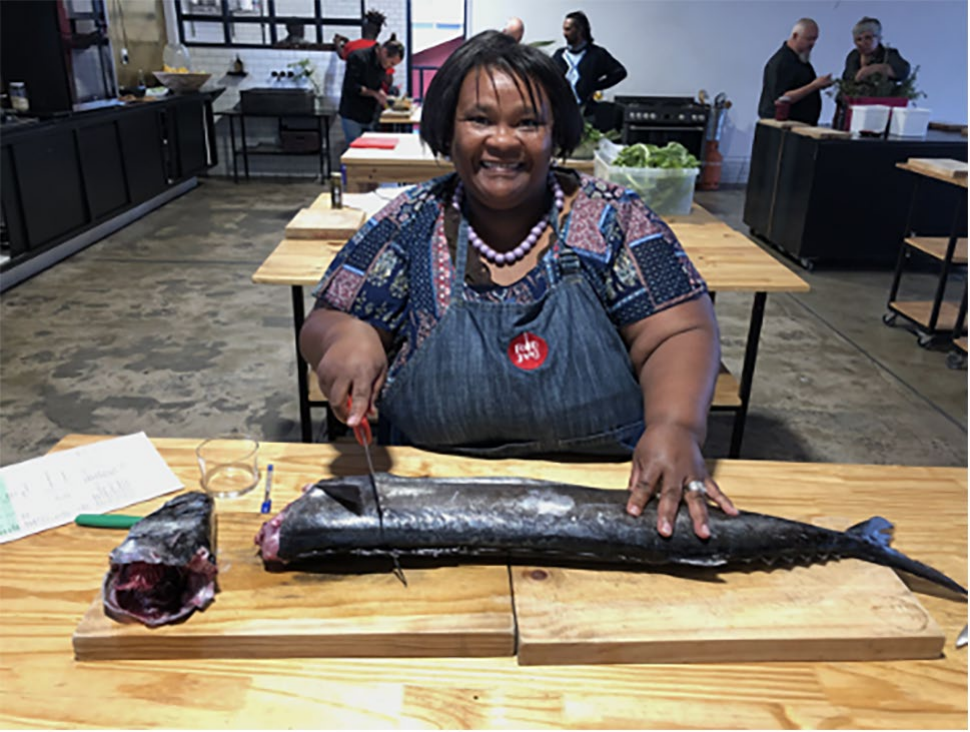
Snoek prepared by workshop participant, Sarah Niemand, during the Food Jam (Photo: Laura Pereira)

Responsiveness to participants' language and food needs was considered as part of the process. Where desired, participants could express themselves in their mother tongue (generally Afrikaans) instead of English, with translation available for those who needed it. Workshop meals were prepared by one of the participants using indigenous coastal food ingredients (Fig. 1). Participants with vegetarian or vegan meal preferences were appropriately catered for.

### Data collection and analysis

The sessions were audio recorded. In addition, three note takers (SBK, JW, and CC) typed notes during the discussions. The facilitators also took notes on clipboards for all workshop participants to see and comment upon. All the notes and audios were compiled into a folder. After each session, the notes were reviewed to fill in gaps. The audiotapes were used to supplement these notes.

The analysis was guided by the topics discussed during the T-Lab to convey the spirit of the conversations and discussions. Much reliance was placed on the flipchart notes that were taken during the sessions, as it was sometimes difficult to make sense of the audio recordings from the workshop. These synthesised descriptions of the discussions, which were marked out in real time with the participants, formed the basis for the sections laid out in the results.

### Case study: South African food system and status of wild food in the Western Cape

The South African food system faces wicked challenges that are globally relevant. The country has one of the world's highest rates of income and wealth inequality and increasing concerns around poverty and unemployment (Stats 2018). Also, in a country that is drought prone and vulnerable to climate change, a more resilient and sustainable food system is imperative. However, it is difficult to shift towards an alternative food system that is simultaneously healthier, more sustainable, and more equitable (Hebinck et al. 2018; Drimie et al. 2018). Within these environmental constraints, South Africa is grappling with socio-economic challenges such as unaffordable healthy diets for many poor households and the continued unequal distribution of land (Greenberg 2015). Most (87%) of all arable land in South Africa is owned by about 35,000 white commercial farmers, while approximately 4 million small-scale black farmers work the remaining 13%, often with little connectivity to the formal food system (Aliber and Cousins 2013).

The limited socio-economic transformation in South Africa means that more impoverished communities, as before and during apartheid, comprise people of colour. These communities have been increasingly exposed to toxic food environments (Kroll 2017). These food environments are characterised by high exposure to Western diet foods that are inexpensive, highly accessible, and heavily marketed, whilst being high in refined carbohydrates, sugars, fats, and salty processed animal-source foods (Brownell and Horgen 2003; Popkin et al. 2012). Furthermore, 'Westernised' diets are culturally disconnected from the local landscape, usually requiring production practices that supersede and dramatically modify the prevailing socio-cultural and environmental context. Therefore, the consolidation of power and profit within the food value chain, food insecurity, and an ongoing nutrition transition (where a Westernised diet supplants traditional food consumption patterns) leave the poor to shoulder the triple burden of malnutrition (May 2017). Indeed, resource-poor communities carry the weight of this toxic food environment on their bodies, which is built upon the low-cost, highly processed foods that are consumed to satiate hunger (Igumbor et al. 2012), with limited consumption of fruits and vegetables (Okop et al. 2019). Ultimately, the combination of markedly high levels of inequality and food insecurity amongst a large proportion of the population leads to an increased burden of non-communicable diseases such as obesity and diabetes, resulting from the transition to cheap and unhealthy diets (Agyemang et al. 2015; Sartorius et al. 2015; Kushitor et al. 2021).

**Fig. 2:**
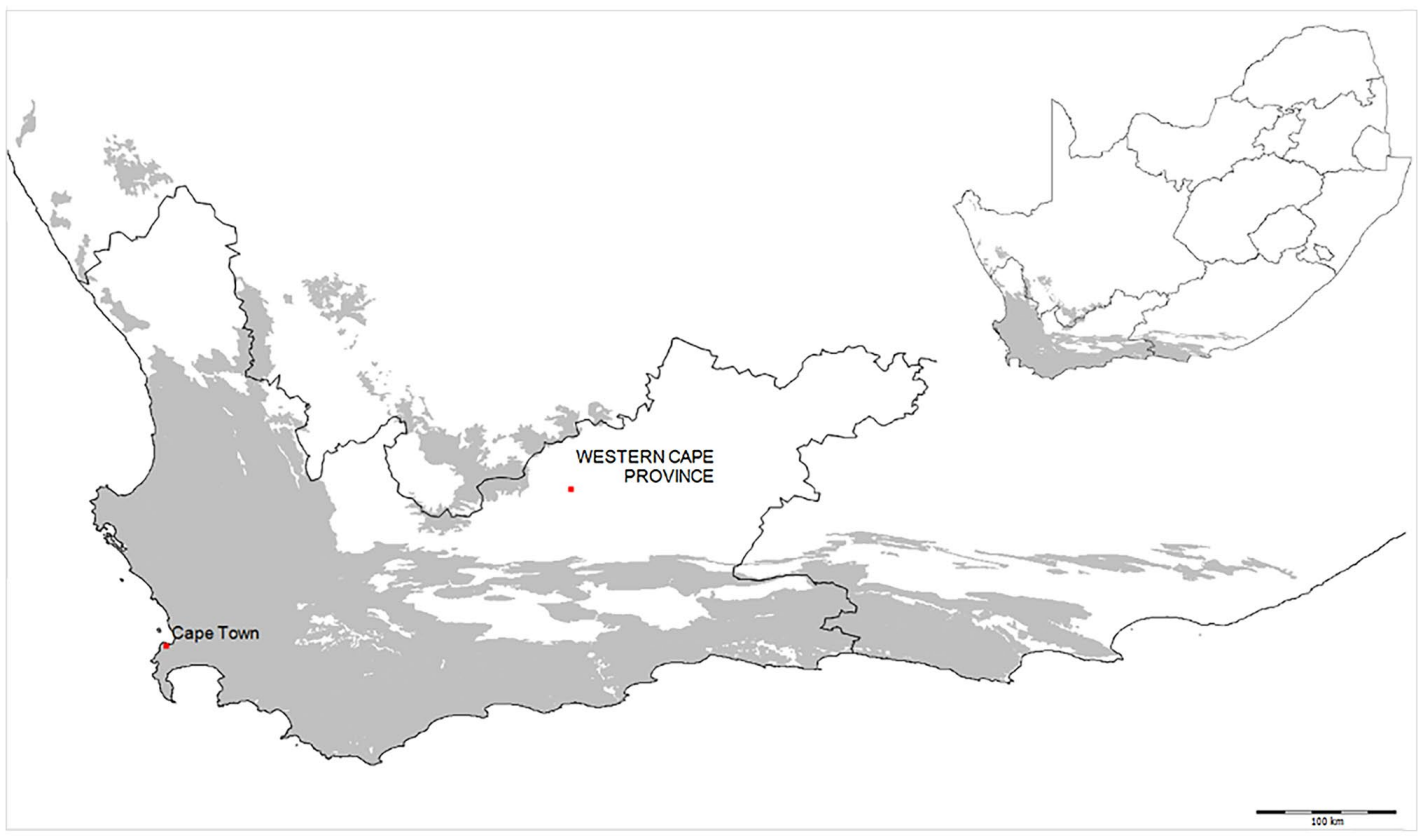
Map of the Western Cape and the extent of the fynbos biome

In this socio-economic and social–ecological context, a fledgling economy in coastal NUS is being driven by chefs of high-end restaurants in the Western Cape (Fig. 2). Ecologically, the Western Cape is embedded in natural fynbos vegetation, named for its fine-leafed plant species. The area is a recognised global biodiversity hotspot with unparalleled concentrations of plant and animal species. Coastal wild foods are a relatively forgotten aspect of the traditional Cape diet. They include many NUS, including marine organisms such as kelp (e.g. *Porphyra capensis* Kützing) and sea lettuce (*Ulva capensis* J.E. Areschoug), as well as terrestrial species such as *veldkool* (*Trachyandra falcata* (L.f.) Kunth), *sandkool* (*Trachyandra divaricata* (Jacq.) Kunth), *soutslaai* (*Mesembryanthemum crystallinum* L.), and dune spinach (*Tetragonia decumbens* Mill.) (Table 2). In addition, species such as sardines (*Sardinops sagax* Jenyns) and anchovies (*Engraulis encrasicolus* Linnaeus), which are fished and reduced to fish meal by industrial trawlers, are also key underutilised species (Isaacs 2016a). This is because fish consumption is reduced to tins of processed fish and rarely includes these species as a fresh food source. Several NUS appearing in the high-end food industry such as Cape lobster (*Homarinus capensis* Herbst), West Coast rock lobster (*Jasus lalandii* H. Milne-Edwards), and scallops (*Pecten sulcicostatus* Sowerby II) are native to the Western Cape and coastal shores that delimit the Cape Floristic Region.

**Table 2:** Neglected and underutilised species found along the coastal zones of the Cape Floristic Region

Plant habitat	Scientific name	Common name	Use
Terrestrial plant	*Agathosma betulina* (Berg.) Pillans	Buchu	Medicinal herb: tea, treatment of internal ailments
Terrestrial plant	*Aloe ferox* Mill*. and* *Aloe vera* (L.) Burm.f.	Aloe	Medicinal: topical treatment, laxative
Terrestrial plant	*Artemisia affra* Jacq. ex Willd.	African wormwood	Aromatic and medicinal herb: respiratory treatment, topical treatment
Terrestrial plant	*Carpobrotus edulis* (L.) N.E. Br,* C. acinaciformis* (L.) L. Bolus*, and* *C. quadrifidus* (L.) L. Bolus	Sourfig	Nutritional fruit snack and medicinal: astringent and mildly antiseptic
Terrestrial plant	*Coleonema alba* (Thunb.) Bartl. and J.C. Wendl. & *C. pulchellum* I.Williams	Buchu	Medicinal: insect repellent
Terrestrial plant	*Dasispermum suffriticosum* (P.J.Bergius) B.L.Burtt	Dune celery	Aromatic herb
Terrestrial plant	*Eriocephalus africanus* L.	Wild rosemary	Aromatic herb: fragrance and respiratory treatment
Terrestrial plant	*Gethyllis* spp. L.	Kukumakranka	Aromatic and medicinal fruit: topical treatment of skin ailments
Terrestrial plant	*Helichrysum pedunculatum* Hilliard & B.L.Burtt and *H. italicum* (Roth) G. Don fil.	Imphepho	Aromatic medicinal herb: cosmetic and internal treatment
Terrestrial plant	*Lippia* spp. L.	Lippia	Aromatic medicinal and culinary herb: repellent
Terrestrial plant	*Lycium ferocissimum* Miers	*Slangbessie*	Fruit snack, topical and poor appetite treatment
Terrestrial plant	*Mentha longifolia* (L.) Huds.	Wild mint	Aromatic culinary herb: respiratory and topical treatment
Terrestrial plant	*Mesembryanthemum crystallinum* L.	Soutslaai	Vegetable and medicinal: oral treatment
Terrestrial plant	*Oxalis pes-caprae* L.	Wild sorrel	Vegetable, carbohydrate source
Terrestrial plant	*Pelargonium citronellum* J.J.A. van der Walt, *P. graveolens* L'Hér., *P. capitatum* (L.f.) L'Hér. ex Aiton, *P. betulinum* (L.) L'Herit.*, or* *P. tomentosum* Jacq.	Pelargonium	Aromatic medicinal herb: fragrance and repellent
Terrestrial plant	*Portulaca oleracea* L.	Pigweed	Vegetable, water source
Terrestrial plant	*Portulacaria afra* Jacq	Spekboom	Vegetable, water source
Terrestrial plant	*Quaqua mammilaris* (L.) Bruyns	*Aroena*	Vegetable, water source
Terrestrial plant	*Salicornia meyeriana* Moss and *S. capensis* (Moss) A. J. Scott	Samphire	Vegetable, carbohydrate source
Terrestrial plant	*Salvia africana-lutea* L. and *S. african-cearulea* L.	Wild sage	Aromatic culinary herb
Terrestrial plant	*Sphalmanthus canaliculatus* (Haw.) N.E. Br.	*Kruipvygie*	Vegetable
Terrestrial plant	*Tetragonia decumbens* Mill.	Dune spinach	Vegetable
Terrestrial plant	*Trachyandra ciliata* (L.f.) Kunth*, T. falcata* (L.f.) Kunth and *T. hirsuta* (Thunb.) Kunth	Veldkool	Vegetable
Terrestrial plant	*Trachyandra divaricata* (Jacq.) Kunth	Sandkool	Vegetable
Terrestrial plant	*Tulbaghia violacea* Harv.	Wild garlic	Aromatic herb
Marine plant	*Ecklonia maxima* (Osbeck) Papenfuss	Kelp	Seaweed
Marine plant	*Laminaria pallida* Greville ex J, Agardh	Kelp	Seaweed
Marine plant	*Porphyra capensis* Kützing	Laver	Seaweed
Marine plant	*Ulva fasciata* Delile and *Ulva capensis* J.E. Areschoug, *Ulva rigida* C. Agardh	Sea lettuce	Seaweed
Marine plant	*Gracilaria gracilis* (Stackhouse) M.Steentoft, L.M. Irvine & W.F.Farnham	Agar weed	Seaweed
Marine fish	*Thyrsites atun* Euphrasén	*Snoek*	Protein source
Marine fish	*Pachymetopon blochii* Valenciennes	Cape bream	Protein source
Marine fish	*Pecten sulcicostatus* Sowerby II	Scallops	Protein source
Marine fish	*Homarinus capensis* Herbst	Cape lobster	Protein source
Marine fish	*Jasus lalandii* H. Milne-Edwards	West Coast Rock lobster	Protein source
Marine fish	*Engraulis encrasicolus* Linnaeus	Anchovies	Protein source
Marine fish	*Sardinops sagax* Jenyns	Sardines	Protein source

Nonetheless, the natural biodiversity that underpins the persistence of fynbos wild edible species is currently threatened by conventional agriculture, urbanisation, encroachment by alien invasive species, and climate change (Midgley and Bond 2015; Richardson et al. 2018; Ntshanga et al. 2021). Furthermore, much of the traditional ecological knowledge (TEK) around the use of NUS in the Cape Floristic Region is considered either lost or declining (Philander 2010; de Vynck et al. 2016a; Botha et al. 2020). Historically, various marine and terrestrial plant species are reported to have been used in everyday life by the San and Khoi peoples for sustenance, health maintenance, and disease prevention (Valente et al. 2014; Bvenura and Afolayan 2015; de Vynck et al. 2016a; Manning 2018; van Wyk and Gericke 2018). Coastal wild foods of the Cape Floristic Region are diverse and include terrestrial and marine taxa, several with dual food and medicinal uses (Table 2). Some of these species are still in use today (de Vynck et al. 2016a).

From a dietary perspective, recent research has focused on the medicinal and nutritional qualities of coastal wild food. For example, Singels et al. (2016) and de Vynck et al. (2016a) have reported a readily available staple source of carbohydrates in the form of many coastal underground storage organs, while coastal wild plants such as *soutslaai* and sour fig (*Carpobrotus edulis*) have been found to have antioxidant and antibacterial properties. *Soutslaai*, in particular, accumulates significant quantities of sodium, calcium, and micronutrients such as iron, while wild sorrel (*Oxalis pes-caprae*) contains high levels of vitamin C (El-Darier 1992; Shevyakova et al. 2003). Aromatic plant species have been used for cosmetic and medicinal purposes as perfumes, soaps, repellents, and even for their antibiotic properties (van Wyk and Gericke 2018). More recently, several of these aromatic species have been trending in elite South African cuisine (Lindow 2017). For example, previously eaten as a snack, the fruits of the sour fig and *slangbessie* (*Lycium ferocissimum*) also appear in current food trends, which may also be extended to include a number of ‘underutilised’ fish species like snoek (*Thyrsites atun*) and Cape bream (*Pachymetopon blochii* Valenciennes) (Willis 2019). Snoek is a significant and highly preferred food option among poor coastal communities. As a result, any possible increase in its use by chefs (e.g. as part of a high-end restaurant trend that seeks to source fresh fish) could reduce the affordability of this high-quality and often cheap source of protein, making it less accessible to social segments that are vulnerable to food insecurity (Isaacs 2013). Similarly, certain kelp species have also received some attention in restaurant menus and in foraging courses offered in the Western Cape. Some examples include sea asparagus (*Salicornia meyeriana*), agar weed (*Gracilaria gracilis* (Stackhouse) M. Steentoft, L. M. Irvine and W. F. Farnham), and sea lettuce (e.g. *Ulva rigida* C. Agardh.).

## Results

Below, we present the core findings that emerged from the rich discussions at the workshop. As this was a transdisciplinary process, and as such had to follow the needs and desires of the participants rather than a strict conventional research structure (e.g. with a focus group), the results are a synthesis of the key themes, concerns, and suggestions that the participants raised. We structure this section first to describe the main threats facing the revival of an equitable coastal wild food system, and then provide examples of where there are opportunities to be leveraged according to the participants.

### Challenges for coastal wild foods

On day one, each of the participants contributed to the discussions from their own unique perspectives. Generally, participants expressed passion and enthusiasm for creating a just food system that espouses identity, environmental conservation, job creation, and food justice. They recounted knowledge from their lived experiences through gardening, advocacy, and cooking demonstrations. An important discussion arose from the perception that food in South Africa could be a very painful topic. For some, this pain had stirred up a passion to work on indigenous food, as a means of making healthy, nutritious food accessible to all. Such efforts included showing people how to grow food with limited resources. To connect people with their environment, some of the lessons focused on the origins of food and how to encourage indigenous people to use indigenous wild ingredients to improve food security. According to one of the chefs, "Food is something I can experiment (with) and introduce to the community to inspire creativity and limitless options" (CHE3). Through their passionate discussions, participants identified a series of threats to a sustainable coastal wild food system. Although the two are closely linked, these include concerns about social and environmental factors, coinciding with our social–ecological framing of the situation.

#### Socio-economic threats

Youth apathy was one of the dominant social threats mentioned by the participants. There was a strong sentiment about a disconnect between the next generation not wanting to get involved in small-scale fishing or farming. Participants cited drugs, crime, gangs, social status, legislation, production cost, and government policies as contributing to this apathy. Youth who are more willing to engage are seemingly up against perceived difficulties that deter them from getting involved.

"Even if your father was a farmer, you cannot have the permit, because there is too much legislation, the cost of production is scary to the youth." (NGO4)

Linked to this was a realisation that youth aspirations for improved livelihoods clashed with their consumer preferences for the type of food, where they purchased it, and from whom. According to the participants, the livelihood aspirations, especially of the youth, cut across economic and racial dimensions, but were negated by consumer choices along the lines of economic and social status. Despite knowing that smallholders have fresh and organic produce at relatively lower prices, consumers prefer to shop from the supermarkets for social identity, class reasons, and acceptance. This social quest reduces the market for smallholders and their profit margins.

"People don't want to buy from smallholders or at the traffic light because it is not as cool as buying from the SPAR." (CHE4)

Associated with youth apathy was the loss of knowledge from previous generations, referred to in the literature as traditional or local ecological knowledge (TEK/LEK). Without generational knowledge transfer, knowledge about edible wild food species and how to use, prepare, and eat them is rapidly being eroded. Even something as fundamental to the local food system as making anchovies from sardines is being lost. Furthermore, it was acknowledged that there was no growth opportunity for sustainable NUS markets, especially when few people know how to prepare the foods or want to eat them.

"It is going to take someone with family histories to make these anchovies. There is a whole process of curing for about 9 months to make anchovies. Anchovy that is not done properly is terrible, horrible, disgusting. Currently, only a few people know how to make it." (FAR1)

Another related aspect was how the participants saw the evolution of capitalism in South Africa as a challenge to sustainability, and a process that disfavours smallholders. Capitalism was said to have produced elite capture of resources, including ocean grabbing and market exclusion for local communities. The system also prevented diversity of service distribution.

"In the current systems, the fishers cannot display their products and be the owners of it. Somebody comes and buy(s) it from them and takes it (to) a fancy market and the face of the fisher is not seen." (NGO5)

Finally, policy is not perceived to be able to handle these threats adequately. The Policy for the Small-Scale Fisheries Sector in South Africa was supposed to enable smallholder fishers access to fishing rights, improve their food security, and address sustainable development, empowerment, and inequality (particularly gender equality). However, the policy is yet to be implemented, leaving many small-scale fishers in limbo, because the government has not indicated when and how the policy will be implemented (see Isaacs 2016b; Gammage and Jarre 2020). At the time of this paper, at the national level, about 22,640 fishers have applied for permits through co-operatives, but only about 10,000 permits will be issued according to the Department of Agriculture, Land Reform, and Rural Development, with the bulk of the permits issued to commercial fisheries and other sectors. This had reinforced a significant concern amongst participants that local fishing companies will export abroad, excluding small-scale fishers from markets as they do not have the resources and time to trade in such spaces.

"All the fish that they are extracting are going for export to European or US markets.

Severe capitalism that does not favour diversity. It likes to conform into the boxes that it has created in order to sustain itself. Everything is big, bigger, biggest." (CHE4)

#### Environmental threats

Plastic pollution was identified as a concern for marine edible species. According to participants, plastics enter the sea through onshore littering by beachgoers and deposition from upstream rivers. Plastics often entangle marine wildlife or are ingested by fish, causing them to choke, become sick, and die. Some fishers reported seeing these impacts on a daily basis.

**Fig. 3:**
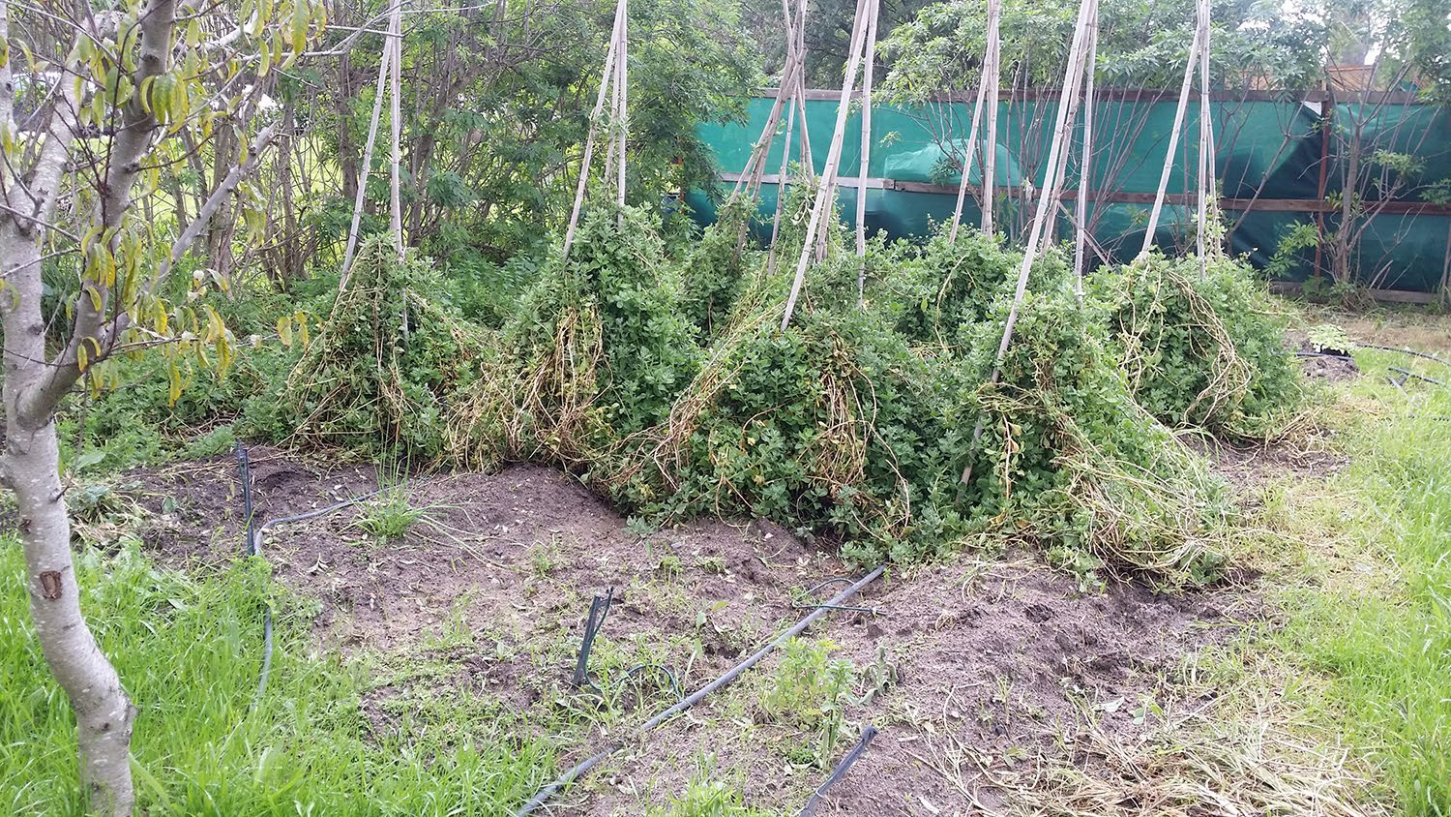
Soutslaai under trial propagation at Stellenbosch University's Agronomy Department (Photo: Chimwemwe Tembo)

While it was recognised that coastal wild foods provide significant sources of food for local households and markets, it was also acknowledged that some practices of wild capture and foraging can have negative environmental impacts if practised unchecked, or by free riders with little vested interest in the persistence of wild species. For example, some fynbos species such as sorels (*Oxalis* spp.) were perceived as endangered by some participants due to their extensive foraging. As noted above, much of the fishing and harvesting is not undertaken by local communities for local consumption, but rather species are targeted only for their commercial value. This is increasingly true for some plant NUS, which are becoming more popular in restaurants, but are not being used by local communities (Willis 2019). Considering the environmental threats facing some plant NUS, it is unfortunate that few resources are invested in research on domesticating these species for use in the modern agrarian economy. A discussion with researchers from the University of Stellenbosch noted that preliminary results from recent planting trials show that local fynbos edibles such as *soutslaai* and dune spinach may be options for local use, with the provision that their production is undertaken in an ecologically sustainable manner (Fig. 3).

### Navigating tensions and challenging the status quo for a more inclusive food system

A strong outcome of the workshop discussions was the commitment of the participants to use local foodways to express and create identities linked to biographic details and geographic positionality. Biographic identities were linked to their reflection on childhood memories about meals and time spent in the garden or on the farm growing their own food. Some respondents shared beautiful memories of long meals with talking, joking, and drinking around the table decked with indigenous foods. The absence of these foods in the market was uncomfortable for them. According to one participant, she was passionate about using snoek to create a Capetownian identity. The motivation for creating these identities was to reclaim lost heritage due to colonialism and apartheid.

"You have to deal with those traumas that are unspoken… It's a very deeply spiritual work. And you don't see it because it's very private also, but it has to be part of what it is that you do." (NGO10)

The conversation on day two revolved on individual next steps. During this discussion, a few difficult issues emerged such as the juxtaposition between, for instance, longtime indigenous food users connected to food through personal heritage, and contemporary food entrepreneurs who do not share those heritage ties. Several participants had years of work and expertise on different aspects of coastal wild foods, while for some participants coastal wild foods were a relatively new concept or something that they had not deeply thought about before. This led at certain points to difficult conversations around next steps that would support existing initiatives or begin something new and a further conversation about what that should be. In particular, deep questions emerged about what it means to be ethical in this domain of work, considering the deep hurt and contestation of whose knowledge this was, and who had rights to that knowledge following centuries of exploitation.

"How do I custodian [other people's cultural heritage through cooking recipes] without taking charge, taking control of it, and taking ownership of it and then turning it into some strange sort of elitist, bastard version that runs away into the distance? …I want to do a lot more of trying to figure out how to achieve that exact balance." (CHE3)

Two participants, both of whom have worked with (and as) indigenous knowledge holders for many years, laid out challenges to privileged participants in the system. They commented about indigenous knowledge exploitation and their inability to benefit from it after it was shared with people in privileged positions. According to them, this exploitation is historical and persistent:

"It is such a precarious space at the moment, because you have a heritage of genocide of indigenous people. …How is it that indigenous knowledge is in non-indigenous, even colonial spaces?” (IND1)

These stark confrontations forced the NUS users in the Cape Town food industry to reflect on their role in the system, whether it perpetuated exploitation or instead enabled local communities to have agency over their local resources. The participants made some of the following commitments: (a) to have conversations with custodians of indigenous knowledge; (b) to let chefs take cooking lessons from custodians of indigenous knowledge; and (c) to let bartenders and restaurants openly share information, with their staff and clients, about where they source their indigenous food, knowledge, and recipes. However, even these commitments were seen as insufficient in building a truly transformed local food system.

"'Oh, we need to tell the story, we need to see the face of the farmer. That's the common thing. But ultimately, what ends up happening is, even if that's your intention, it doesn't go very far because the reality of the farmer or producer or the person you're trying to support doesn't really change. Not in the long run." (NGO10)

The discussion then turned to questioning whether it was possible to truly honour and acknowledge (as distributors and chefs) all the people whose knowledge has enabled the cultivation and use of these plants. This clarified the need to move away from generating the very short-term solution of financial exchange and support to a far more radical question around how to support the legal struggles for people whose rights have been undermined for generations. This challenge opened up the space for a radical debate about the role of food and our diets as deeply political. One participant challenged others to use the T-Lab as a space "where people can stick in. Because if people who hold the kind of clout that people in this room do, are able to push through the political agenda a little bit more, that would change things quite drastically. Or potentially could change things quite drastically" (IND1).

Interestingly, these exchanges highlighted the issue of trust between the participants. Some participants were reluctant to commit to personal next steps until they could see commitments from others who were less vulnerable, and once that trust was there, they would be willing to work more collaboratively. This iterated that trust in a contested system, such as that of food, takes time to develop and that issues around power dynamics needed to be carefully considered: "I…feel that this space is not safe enough for me to talk about my deep reflections" (Anonymous^1^). This galvanised other participants to commit to taking some actions to make the space feel safer by making sure that they are present in the cause, asking questions, and taking time to listen to others, not assuming what the answers will be. An ‘open mind and an open heart’ were recognised as critical to facilitate that listening.

## Discussion

“Results” highlighted some of the critical challenges facing the equitable invigoration of a coastal wild foods system in the Western Cape, but it also brought to light the significant interest in undertaking the necessary work. Here, we situate the discussions raised in the T-Lab within broader themes of how coastal wild foods could contribute to more sustainable and healthy diets, how concerns over knowledge of these foods can be managed more inclusively, and what are the lessons learned for food policy. We conclude with a reflection on the overall T-Lab process.

### Coastal wild foods as a cornerstone of sustainable and healthy diets: potentials and pitfalls

Sustainable and healthy diets are dietary patterns that are accessible to all, socially and culturally acceptable, safe, nutritious and desirable, and have low environmental impact (FAO/WHO 2019). At this stage, more information needs to be generated to advocate for the stronger incorporation of coastal wild foods into local diets, such as identifying their nutritional properties. Free access to sound nutritional and food safety information in the form of dietary guidelines that include medicinal, antifungal, and antimicrobial properties is essential to building public trust in coastal wild foods. For example, extensive research has been conducted to assess the nutritional content of a close relative of dune spinach, New Zealand spinach (*Tetragonia tetragonoides* (Pall.) Kuntze), which was found to have good nutritional value, essential amino acids, vitamin C, various minerals, and high antioxidant activity (Wheeler et al. 1939; Jaworska and Kmiecik 1999; Słupski et al. 2010; Neubauerová et al. 2011). This is a strong indication that many similar coastal wild foods are likely also to exhibit good nutritional content. Inadvertently, this may also increase their role in combating obesogenic and toxic food environments (Agarie et al. 2009; Bouftira et al. 2012), such as those observed in many parts of South Africa (see above). However, nutritional and food safety analysis can be costly, thus requiring researchers to advocate that existing funding instruments facilitate the investigation of coastal wild food properties as part of co-designed processes that include women and indigenous knowledge holders in the discussion on sustainable consumption.

Coastal wild foods can also have a substantial role in contributing to healthy diets due to their potential in creating environmentally sustainable food systems (FAO/WHO 2019). The adaptation of coastal wild foods to their environment makes them well suited for developing agroecological production systems that facilitate the production of sufficient crop quantities while efficiently utilising already limited resources such as soil, water and nutrients (El-Darier 1992; Cecílio Filho et al. 2017). For instance, due to their halophytic nature, some leafy coastal vegetables (e.g. dune spinach, *soutslaai*) have been found to be well adapted to drought and high-temperature stresses, with a significant tolerance to the Western Cape's saline and poor quality soils (Bartels and Sunkar 2005; Yousif et al. 2010; Visscher et al. 2018). In addition, the cultivation of coastal wild foods can become a tool to improve crop and soil quality and increase agricultural water use efficiency in regions experiencing scarcity of water resources (Bohnert and Cushman 2000; Hasanuzzaman et al. 2014; Tembo-Phiri 2019).

Therefore, as noted in the T-Lab, there may be good economic incentives and potential for producing coastal foods through urban horticulture for both consumption and sale. However, any potential coastal wild plant cultivation would need to pre-empt an increase in foraging pressure, which might become unsustainable as more people engage in foraging. Reviving the use of NUS, in an ecologically sound and socially inclusive economy, may offer an opportunity to contribute towards food sovereignty and a more ecologically appropriate agriculture that results in improved dietary diversity, while simultaneously reversing the loss of TEK in the Cape Floristic Region. A good example of this was demonstrated by a Zambian researcher T-Lab participant who brought a taste of chikanda to share at the workshop.

**Fig. 4:**
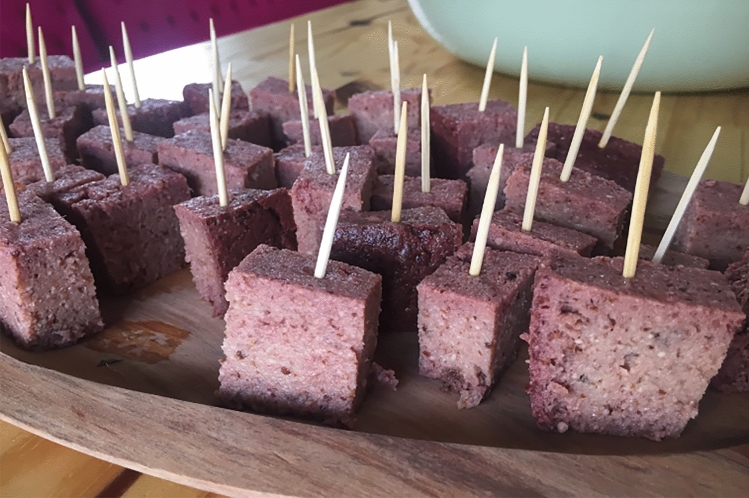
Chikanda cubes at the T-lab: a Zambian wild food delicacy. Note: chikanda is a soft textured meat-like product prepared in a 1:1 ratio of finely ground orchid tubers and peanut flour. Peanut flour is added to boiling water containing salt, baking soda, and occasionally chilli powder. Chikanda powder is then added to thicken and bind the mixture giving the product a gelatinous texture, similar to that of polony (Kaputo 1996) (Photo: Carolyn Cramer)

Chikanda is a remarkably meat-like plant product known as African polony or Zambian sausage (Kaputo 1996). This nutritious snack and delicacy is eaten at local markets, restaurants, and on special occasions in small portions such as small cubes (Fig. 4) (Veldman et al. 2014). Chikanda is made from the tubers of different species of several endangered terrestrial orchid genera (Veldman et al. 2018). Davenport and Ndangalasi (2003) estimated that between 2.2 and 4.1 million tubers are harvested per year for this purpose, with species of *Disa, Satyrium*, and *Habenaria* and, more recently, the *Brachycorythis* genera being the most popular. Tanzania's Southern Highlands are a major source of these orchids, with at least 85 species that have been identified, while 35 marketable tuber species have been identified in Zambia (Veldman et al. 2018). Although there have been many ethnobotanical studies and collections of herbarium orchid specimens in the past (Nyomora 2005; Veldman et al. 2017), population density studies are relatively limited, making it difficult to assess the impact of harvesting on wild populations. Nevertheless, chikanda orchids are harvested, traded, sold, and consumed by local communities and the diminishing quality and quantity of the species used to support this food-related use will make it increasingly rare without the establishment of cultivation protocols (Lalika et al. 2013; Hinsley et al. 2018). In a sense, chikanda has now transformed from being a traditional food produced and eaten in northern rural areas (where foraging orchids was a major aspect of local ecological systems), to a lucrative source of income with an established local value chain that is supported by orchid trade between Zambia and Tanzania, Angola, Malawi, and the Democratic Republic of Congo (Kasulo et al. 2009; Veldman et al. 2018). Despite recent attempts at propagating these edible orchids, they have only started after decades of wild harvesting and urban commercialisation (Davenport and Ndangalasi 2003). The transformation of chikanda from a wild harvested product to a lucrative income source has driven micropropagation trials currently underway at Copperbelt University in Zambia, but cultivars are yet to be developed. The chikanda case outlined above offers a stern warning about the potential pitfalls of commercialising NUS into a status symbol or popular food item, without having the available means for supply from the cultivation stage.

The growth of a NUS food economy in the Western Cape area is not a straightforward solution for sustainable diets and poses its own risks. One relates to the potential ecological damage from wild harvesting, while another risk relates to the potential shift to monocrop cultivation in a region where several vegetation types and microbiomes are already threatened. A third risk is further entrenching of historically established power dynamics and commercialising traditional knowledge without local community buy-in (Willis 2019). So far, the commercialisation of NUS has not contributed to equity in the coastal food system, but has, instead, created new forms of power elites within the communities, whilst “exporting” nutritionally rich foods to service wealthier consumers (Willis 2019). While in certain cases food has "proved to be a useful tool to break down, or at least circumvent power relationships and help gain a deeper understanding of place and culture,” (Haider and van Oudenhoven 2018: 14), there is also a risk of reproducing power relations. For example, the edible insect movement has been characterised as having potential to promote ecological stewardship, bio-cultural diversity, and food security (Evans 2014), but has failed to deliver large-scale impact in the reinvigoration of NUS. Instead, it has often reproduced power relations in globalised food systems as much as challenging them, with little benefit accruing to those whom it claims to empower (Müller et al. 2016).

Therefore, it is important to ensure that the potential benefits of building a NUS economy in the Western Cape food system do not further entrench the other inequities prevalent in the South African context. This is the critical context within which the coastal wild food T-Lab was situated, and what emerged from the T-Lab is the stark reminder that despite all of their benefits, there is a great potential for capture of NUS by powerful elites. It is of utmost importance to ensure that the economic benefits expected from the increased use and production of NUS are equitably shared with those who have held the relevant knowledge of these species for centuries. In this sense, it is critical that the focus should not just be on commercialisation and cultivation for economic and environmental needs, but also for developing a more inclusive and equitable food system.

### Knowledge and equity in enabling a more inclusive food system

The inclusion of TEK and its meaningful integration with scientific knowledge were raised as important first steps towards developing a sustainable and equitable food system based on indigenous foods in the Western Cape region. The T-Lab highlighted that there was a fledgling NUS economy driven by high-end chefs. With access to niche markets, these actors promote among their clients the use and value of NUS as "exotic" sought-after products. At the same time, as indicated in the T-Lab, there is a significant informal NUS use economy amongst poorer people, many of whom value these species as part of their cultural heritage, but nevertheless operate at the margins of formal food systems. While much of the TEK around the use of NUS in the Cape Floristic Region is considered lost, recent studies attest to a sustained knowledge base amongst NUS users in different communities across the biodiversity-rich phytoregion, especially amongst residents with generational ties in these areas (de la Fontaine and Malgas 2013; de Vynck et al. 2016a; Malgas et al. 2018; Tembo-Phiri 2019; Topp et al. 2021). By including TEK, it can possibly prevent the further exclusion of the most marginalised members of the local communities (and their local knowledge), instead facilitating equitable ways to connect diverse cohorts of contemporary users and knowledge holders.

The persistence of wild NUS is central to issues of food justice, job creation, livelihoods, and business. Several endemic NUS in the Cape Floristic Region have started to appear in the high-end food industry, in a region where terrestrial biodiversity is already threatened by various anthropogenic activities, including urbanisation and conventional agricultural production practices. Similarly, many of the marine species harvested from the ocean, tidal, and inter-tidal zones are also affected by pollution, and secondary waste from those activities that threaten terrestrial environments. Regrettably, only 1% of Cape Strandveld fynbos and 2% Cape Sand fynbos remain in the natural landscape (City of Cape Town et al. 2010), although both are the naturally delimited vegetation types that support the growth and persistence of wild endemic plant populations. The persistence of NUS and other food species in the wild is therefore also threatened as human activities increasingly encroach on these natural habitats. Considering the above, we argue that the conservation and management of these valued resources are best achieved by leveraging knowledge (scientific and traditional) and policy for collective action. However, their conservation competes with other pressing social needs for housing, recreational space, and industrial development, which drive many of the threats to biodiversity.

A food system based on indigenous coastal species necessitates an ethic of ecological and social integrity amongst all actors and across the entire value chain, if it is to achieve its objectives of sustainability. Framing food systems as social–ecological systems, with TEK as a central element, creates an opportunity to foreground the synergies that do exist between local NUS users and their natural environments (Guerrero Lara et al. 2019). The knowledge of local NUS users about ecosystem goods and services has been shown to inform wild harvesting and agricultural production of some indigenous crop species in the Cape Floristic Regions, such as rooibos (Malgas et al. 2018). This highlights the role that TEK can play for the conservation of scarce natural and agricultural resources in a biodiversity hotspot, where indigenous food production is only one of many land-use demands.

Despite growing recognition of the value of TEK in advancing indigenous food systems, there are also knowledge gaps that hinder their establishment. A lack of knowledge on food harvesting, processing, preparation, and nutritional value, for instance, limits the diversity of foods available to achieve food security (Akinola et al. 2020). This can leave coastal communities vulnerable to exploitation, exclusion within the food system, and the commodification of the foods. It might also limit market development for other alternative user groups such as consumers further down the value chain. In local knowledge systems, knowledge gaps around food systems are attributed to disruptions in generational knowledge transfer, which is one of the most important ways through which indigenous knowledge systems are retained (Aswani et al. 2018). Conversely in conventional scientific knowledge systems, knowledge gaps are attributed to the historical exclusion of TEK from scientific endeavours, the strong research focus on commercially important food commodities (to the exclusion of indigenous crops) and, as a result, a lack of knowledge about the ecological and agronomic parameters for wild harvesting and production. These knowledge gaps are best addressed in ways that complement these different knowledge systems, in that synergies are likely to lead to sustainability outcomes better than what each knowledge system could contribute on its own (Tengö et al. 2014). A sustainable and equitable indigenous food system is essentially a social justice issue, and as such has much to gain from synergic knowledge co-production amongst the diverse NUS users in the Western Cape foodscape.

With coastal wild foods still largely unknown by the average citizen, visual cooking demonstrations and nutrition education campaigns that develop consumer empowerment would be necessary to make these foods relatable, while laying the basic foundations of how they can be utilised at home (Lindow 2017; Tembo-Phiri 2019). Considering the many cultures and communities in South Africa, coastal wild food preparation would need to acknowledge and cater to these differences to make them more appealing to diverse consumers. With about 49.2% of South Africans earning less than 1,200ZAR/month (100USD), it is imperative that coastal wild foods do not remain in the niche market, but rather become available and affordable to all, especially resource-poor communities. Nevertheless, it is also important to consider and address power dynamics and inequities before further developing the NUS market in the Cape Floristic Region, so that it does not adversely affect local communities who previously relied on these foods or relevant TEK holders. Inclusive policy measures will be critical in ensuring the equitable development of this NUS market to create a more sustainable and healthy food system. However, as the T-Lab outlined, this is not a simple task given the historical erosion of knowledge and the lack of trust from years of oppression of marginalised groups.

### A complicated role for food policy

Although coastal wild foods may help to mitigate food insecurity by providing access to highly nutritious food, the foraging of indigenous coastal edible greens is often constrained by socio-economic biases, historical legacies, and incoherent policies (Davenport et al. 2011; Shackleton et al. 2017). This perception was reinforced through the discussions during the T-Lab. While policy coherence refers to the coordination of policies across sectors to support food security, the lack of policy coherence might contribute to the ineffectiveness of food security initiatives (Brooks 2014). Little is known about the impact of policy on foraging for indigenous food plants in South Africa, but there is a clear mismatch in existing policy surrounding fisheries.

For example, the western South African coastline is one of the most productive large-scale marine ecosystems on the planet (Blanke et al. 2009), which is rich in marine and other resources, contributing significantly to national economic development (WWF 2011). In addition, the fishing industry provides about 60,000 tonnes of seafood each year and employs both large-scale and informal sector workers. Yet, access to fishing rights and coastal resources remains a political challenge, and it is for this reason that small-scale fishing challenged the rights allocation in 2004 with the class litigation of “Kenneth George and Others vs. the Minister of Environmental Affairs and Tourism” for their rights to livelihood and food security (Isaacs 2016b). In an out-of-court settlement, small-scale fisher representatives and fisheries officials drafted a new small-scale fisheries policy for South Africa (Isaacs 2016b). In this policy, it is enshrined that small-scale fishers' “use of marine living resources on a full-time, part-time or seasonal basis in order to ensure food and livelihood security… also means the engagement (by men and women) in ancillary activities such as, (pre and post harvesting, including preparation of gear for harvesting purposes), net making, boat-building (beneficiation, distribution and marketing of produce), which provide additional fishery-related employment and income opportunities to these communities” (Republic of South Africa 2012: iv).

This policy seeks to secure social and economic justice for this sector, but almost ten years after the policy was released, many small-scale fishers viewed the implementation as a challenge. As a result, lack of trust between parties remains, as commercial entities still capture most of the benefits. To promote inclusivity in policy implementation, accurate information is required to create food policies that are unique to communities in terms of their food security and nutritional needs (Branch et al. 2002; Gammage and Jarre 2020). It is also critical that not only the development of the process be participatory, but that there is political will to ensure its continuous inclusivity through/during implementation. Unfortunately, the political will to ensure localisation of marine and coastal indigenous food plants is currently missing in all spheres of government—local, district, and provincial. A key conclusion from the T-Labs is that food policies should be tailored around experiences, rather than being highly centralised, and that this learning needs to be taken into the wider NUS policy space to ensure localisation and sustainability of the edible species.

As much as synergies were sought, the workshop also pointed to the tensions between urgent social needs and constrained environmental limits. For example, the implications of foraging indigenous species for a growing market may not be viable before these indigenous edibles are cultivated due to the sustainability implications of foraging for the protected fauna and flora in the fynbos landscape and surrounding coast. On the other hand, the work towards developing an equitable and just food system may also catalyse a social reconnection amongst actors and between society and the environment. Navigating these various trade-offs is a complicated role for policy.

### Lessons learnt through the T-Lab

In essence, the T-Lab sought to create an opportunity to enter dialogue around a contested topic with a range of stakeholders having different experiences and perspectives. Through this process, ideas about how to engage in this contested space emerged. A key lesson learnt from the T-Lab is that a facilitated dialogue can be an effective method to help identify multiple solutions and support change strategies, but also that in itself this is only part of the process (Drimie et al. 2018). Building trust and commitment is necessary to take these ideas into reality where they can be tested and adapted. The T-Lab process was effective in framing the challenge and building some trust and understanding among stakeholders that could sustain action, but far more is required to strengthen processes to address more effectively the challenge. The T-Lab successfully enabled the beginning of a process in which stakeholders from different sectors and backgrounds (and many with different perspectives) could begin to build a shared understanding of the challenge and begin to work together to creatively co-design novel and testable innovations.

Overall, the T-Lab proved to be an appropriate method for approaching this contested topic, but it is an ongoing intervention that would require continuous support and mobilisation. This can be difficult to achieve through academic research funding. Two different sets of funding have been used to host relevant T-Labs in the Western Cape thus far, which has allowed for a longer-term process to be established and social capital to be built between the researchers and participants. As funding is never guaranteed, the investment of time, money, and effort could dissipate over the long term if alternative mechanisms are not found. A move towards eventually establishing a community of practice that can be self-sustaining would be a significant longer-term achievement, and there should be an emphasis on this as discussions proceed. It seems that inroads are currently being made towards the establishment of such a community of practice under the auspices of the NGO Local Wild and in collaboration with the Sustainability Institute at Stellenbosch University. It is nevertheless important to be open and honest with participants from the beginning about this to manage expectations. However, it also speaks to the need for larger reconfigurations of the academic funding sphere, as it moves towards emphasising transdisciplinary work and knowledge co-production. Funding mechanisms need to recognise that such processes take time and are emergent, that trust is not earned overnight, and that often the outcomes of such processes are often not what was originally intended, but may be more important in the long run (Scholz 2017).

Similarly, building the skill sets of facilitators who are able to design and implement such transformative spaces that can hold conflict, whilst allowing participants to feel safe enough to articulate their viewpoints, is an important capacity gap (Marshall et al. 2018). As reflected by Pereira et al. (2020b) on the different capacities needed for transformative space research, it is important to develop these kinds of skills and document the relevant processes so that lessons can be learnt and shared in other contexts. Sharing such insights and reflections with the broader sustainability science community which is invested in deep transdisciplinary research can hopefully start to foster a space where knowledge can be shared effectively, so that future attempts at such endeavours will be able to build on what has been implemented and succeeded before.

One of the limitations of a T-Lab as a process is that it can be very difficult to record the proceedings and discussions for later analysis. Taking notes during such interactive processes on a tablet or notebook was seen as a distraction to participants, as "being observed" can hinder people's ability to speak freely. The pace of discussions might also cause the observer to miss important aspects of the event. Despite these limitations, there was a considerable body of information and knowledge captured, from which to draw for the analysis presented in this paper. A potentially effective way to capture some of the proceedings would be to undertake some semi-quantitative ranking process with the participants around the various threats and interventions. The key is to ensure that this does not detract from the rich narratives and experiences that can unfold during the course of the two days. Overall, this is an ongoing case of learning by doing of how to capture important information, whilst allowing the process to unfold as the main intervention. Further reflections on how similar processes have been able to navigate this difficulty would be a welcome addition to the growing transdisciplinary literature, especially the literature on how to undertake transdisciplinary research with care (Sellberg et al. 2021).

The process of transdisciplinary research needs to also judiciously confront upfront the intersectionalities of the group (e.g. in terms of race, class, gender, rank, language, education, wealth) (Takeuchi et al. 2020). A possible way would be to bring together people from different backgrounds with different agendas before the workshop, but it would need time and resources to get the group to agree to common values and vision and ensure that knowledge sharing would not be appropriated. Continued dialogue between the diverse participants may expedite this connection over the long term, but it is also important to note that not each T-Lab participant might be feeling satisfied with the processes, especially around challenging topics. Continued dialogue might facilitate smoothing out some of the lingering conflicts. As researchers, we need to acknowledge that challenging topics require time, particularly when there are diverse perspectives. Finally, when co-designing the process, it is vital that the knowledge is co-owned, and that the outputs are also co-produced with those who are interested.

## Conclusion

The T-Lab on coastal wild foods reported in this study brought a diverse group of stakeholders together to engage deeply on how a more sustainable, healthy, and equitable food system centred on local NUS might emerge. As an emerging tool of knowledge co-production around food system transformation, this T-Lab opened up not only surface concerns, but deeply embedded issues around land, race, history and culture that need to be grappled with if a better food system is to emerge in South Africa. T-Labs continue to be an emerging concept, and this application within the Western Cape is a contribution to this growing knowledge base in the global South about how to initiate spaces of co-production aimed at equitable sustainability transformations. This T-Lab, the third conducted in the Cape Town area, aimed to connect a diverse range of stakeholders through dialogue and guided reflection within the wild coastal food space, strengthening connections across sectors and fields. By convening a group of people who recognise the potential of coastal wild foods for contributing to the Western Cape food system, the T-Lab was able to build relationships between a key set of stakeholders in anticipation of future opportunities to disrupt or change the dominant system. This emerged through a process that was able to foster a deep awareness of the ethical context, in which the project is situated.

It was evident that the T-Lab provided a platform where participants could learn and share with one another, while delving into the threats and opportunities related to coastal wild foods. It created a space for relationships to be formed and strengthened, collaboration to be envisioned, and a set of principles and action steps to be discussed by a group that can often be marginalised from core decision-making processes. A critical insight is that whilst developing a local food system around coastal NUS could have far-reaching health and sustainability benefits, the equity implications of whose knowledge is being leveraged and who has control over the transition are critical areas that food policy should tackle. This is vital for similar work undertaken in other parts of the world to appreciate (especially where the inequities might not be as stark as they are in South Africa), but are nevertheless important to consider. It is also critical for policymakers to take note of these potential pitfalls in innovations for food system transformation, and for them to become embedded in such processes with the intention of gaining a deeper understanding of the complexity confronting such policy environments.

## Footnotes

1 Affiliation is not disclosed for confidentiality.
